# Using Haptic Feedback in a Virtual Reality Bone Drilling Simulation to Reduce Plunge Distance

**DOI:** 10.7759/cureus.18315

**Published:** 2021-09-27

**Authors:** Miles W Benjamin, Omar Sabri

**Affiliations:** 1 Trauma and Orthopaedics, St George's University Hospitals NHS Foundation Trust, London, GBR

**Keywords:** simulation medicine, virtual reality, haptic, higher education medical training, drilling, simulator, oculus

## Abstract

Background

Bone drilling is a procedure that demands a high level of dexterity, fine motor skills and spatial awareness from the operating surgeon. An important consideration when drilling bone is minimising soft tissue damage. There are numerous causes of drilling associated soft tissue injury, of which most concerning is drilling into the tissue beyond the far cortex as unseen injury can occur. This is known as plunging.

Objectives

The objective of this study was to evaluate the impact of haptic feedback in virtual reality (VR) simulation-based training. The acquisition of drilling skill was assessed by changes to their drill plunge depth.

Study Design & Methods

The participants in the study were medical students, doctors and biomedical scientists. Participants were randomly allocated into two groups. One group had simulation with haptic feedback as part of their VR simulated learning, whereas the second group undertook the same VR simulation but did not receive haptic feedback during the simulation. Following completion of the simulated bone drilling protocol, a bone drilling exercise took place. Each participant was allowed to drill a synthetic tibia bone five times and then the plunge depth was measured. We quantified outcome in the form of plunge depth.

Results

There were four participants in each group. The average plunge distance in the group who were able to practice with haptic assisted VR simulation was 46mm (range: 37-56mm), the average plunge distance in the non-haptic group was 79mm (range: 44-136mm). Results showed an average reduction of 33mm in plunge depth from users in the haptic group compared to the non-haptic group.

Conclusion

Bone drilling simulation with haptic feedback may be an effective simulator of the motor skills that would be required to perform this action on a live patient. The study results suggest that there could be a reduction in soft tissue damage for users trained in VR simulations with haptic feedback.

## Introduction

Virtual reality (VR) is a tool that allows users to be transported to a different environment, one that may be a replication of a real-life scenario in order to develop skills. Due to modern-day technological advances, the use of VR technologies within medicine has increased throughout the years. Initially used within surgery to serve as an aid for preoperative planning [1,2], it has now transformed into more sophisticated devices.

In order to create an environment with VR that is multi-sensory, a setup that incorporates the collective use of a haptic device, headphones for audio stimulation and a head-mounted visual display is commonly being used to fully immerse users in a simulated environment [3]. The haptic device applies defined force feedback on the users' handling tool. This allows the user to feel the virtual objects that they see through the VR headset, which produces as close to real-life sensations as possible.

To be classed as being surgically competent, one requires the combination of surgical technique, experience and strategy. The COVID-19 pandemic has caused a dilemma for surgical educators delivering effective training. The pandemic has led to significant training disruptions for trainees of all levels. The Royal College of Surgeons surveyed 970 surgeons training across the United Kingdom. Two-thirds of surgeons indicated that the lack of elective surgery meant that there were fewer opportunities in training. Gaining time in the operating room has acted as a significant barrier to training [4]. The difficulty in obtaining sufficient learning experiences makes performing procedures increasingly more difficult for trainees. If one is not surgically competent, then surgical-related complications may be greater, putting patients at risk [5].

This is where VR environments can create a training opportunity for surgeons. VR technology has allowed trainees to overcome the learning curve of various surgical procedures [6]. The benefit of training within a simulated work environment is that it is safe and allows users repetition of surgical skills without causing any harm to the patient. VR simulators have incorporated the use of haptic feedback devices in their models. The science of haptics allows man-made controllers to have a realistic sense of touch, which is performed through force-feedback depending on the structure that the operator is handling. Within a simulator, this occurs when the operator pushes or pulls an object, and the resistance felt increases until yield pressure is achieved [7].

Waterman et al. studied the use of simulation as a method of improving surgical skill in arthroplasty [8]. Participants received either a VR simulation curriculum or a standard practice curriculum without VR simulation. The study found that the group receiving the surgical simulation curriculum performed better when assessing participants' procedure times and handling of the instruments.

Cannon et al. studied the use of a knee arthroscopy VR simulator for residents [9]. Their results suggested that the residents receiving VR simulator training prior to the live procedural skill showed a greater skill level in the operating room when compared to residents who did not receive VR simulator training.

Further emphasis has been placed on motion metrics and data analysis of the precision with which the input device is moved. Studies have shown that the skill level of surgeons can be assessed using motion analysis during procedures [10-12]. Genovese et al. evaluated the efficacy of motion tracking in determining surgical skill level [13]. They demonstrated that skilled surgeons have more controlled movements and these movements are quantifiable by a computer.

In orthopaedic surgery, cortical bone drilling is a core skill that requires a high level of dexterity, fine motor skills and spatial ability from the operating surgeon. The purpose of the task is to drill through the entirety of the width of the bone and stop the drill bit before it can damage the soft tissues or important anatomical structures on the other side of the bone. The distance the drill bit travels past the far cortex is called the plunge distance. This may be considered a form of clinical and technical error. This skill is typically practised in the operating room on live patients and plunging error may lead to soft tissue damage and unseen injury [14].

The purpose of this study was to investigate how the presence or absence of haptics affected the plunge distance in the drilling of a synthetic tibia (Sawbones®, Vashon, Washington, United States).

## Materials and methods

Participants

Eight participants (mean age = 29.7 years) were included in the study, with no experience of being on a surgical training programme. Participants self-reported having none to low levels of experience using a drill in a surgical theatre. Participation was voluntary, and the study was advertised by email and word of mouth at St George’s Hospital and St George’s University London. Participants provided written informed consent prior to participating in the drilling study. The participants recruited were medical students, junior doctors and biomedical scientists associated with St George's University London or St George's Hospital London with no experience of the surgical training programme within the United Kingdom.

A pre-procedure questionnaire was completed by participants (Table 1). It was used to assess participants’ hand dominance and operative experience (observed, assisted, primary surgeon). In addition, the questionnaire assessed experience using a domestic drill, experience of VR, use of a drill in the operating room, use of an orthopaedics simulation platform and use of console-based video games.

**Table 1:** Pre-study questionnaire for participants

Tibia Drilling Study Pre-Questionnaire
Demographics
Course/training programme:
Year or level of training:
Dominant hand:
Please estimate how many operative cases (if any) you have observed or assisted in orthopaedic surgery:
Please estimate how many operative cases (if any) you have performed as primary surgeon in orthopaedic surgery:
How would you rate your prior experience of the following? (Please use the following scale None - 0, Little - 1, Low to Moderate - 2, Moderate - 3, Moderate to High 4, Extensive - 5)
Using a domestic drill
Experience of VR/immersive games
Use of a drill in the operating room
Use of an orthopaedics simulation platform
Use of PC or console-based games
How would you rate your knowledge of the following? (Please use the following scale None - 0, Little - 1, Low to Moderate - 2, Moderate - 3, Moderate to High 4, Extensive - 5)
Anatomical structure, features and landmarks of the Tibia?
The safe, operational and surgical use of an orthopaedic surgical drill?
Any further comments?

A post-procedure questionnaire was also completed by participants (Table 2). It looked at participants' thoughts on the study and its use of haptic feedback simulation. 

**Table 2:** Post-study questionnaire for participants

Tibia Drilling Study Post-Questionnaire
All statements are rated using the following scale: Strongly Disagree – 1, Disagree – 2, Neither agree nor disagree – 3, Agree – 4, Strongly Agree
In relation to your assigned simulation experience rate the following statements to the best of your knowledge. Please rate according to your perceived experience, even if you have limited real-world orthopaedic exposure:
I enjoyed using the surgical simulator
I found the simulation a valuable learning experience
The surgical instruments and their actions looked realistic
The instruments and their actions felt realistic (i.e., hand interface, weight, force feedback)
The instruments and their actions sounded realistic
The bone and soft tissues appeared realistic
The simulated scenario represented a realistic clinic scenario
The possibility to have haptic (force-feedback) would be/is a crucial element when training this task
I would use this simulator if it were made available to me
Simulation should be included as part of surgical training
The simulator helped improve the following:
My theoretical knowledge of how to do the procedure
Trained me how to use the instruments
Trained me how to avoid over drilling or damaging structures with drill
Trained me how to recognise when to stop drilling/complete the procedure properly
Would help improve my clinical outcomes when operating on patients
Increased my confidence in the safe use of surgical tools
Increased my competence in the safe use of surgical tools
Regular use of a surgical skills training simulation (such as this drilling exercise) would be valuable to surgical training
Any further comments?

Procedure

**Figure 1:**
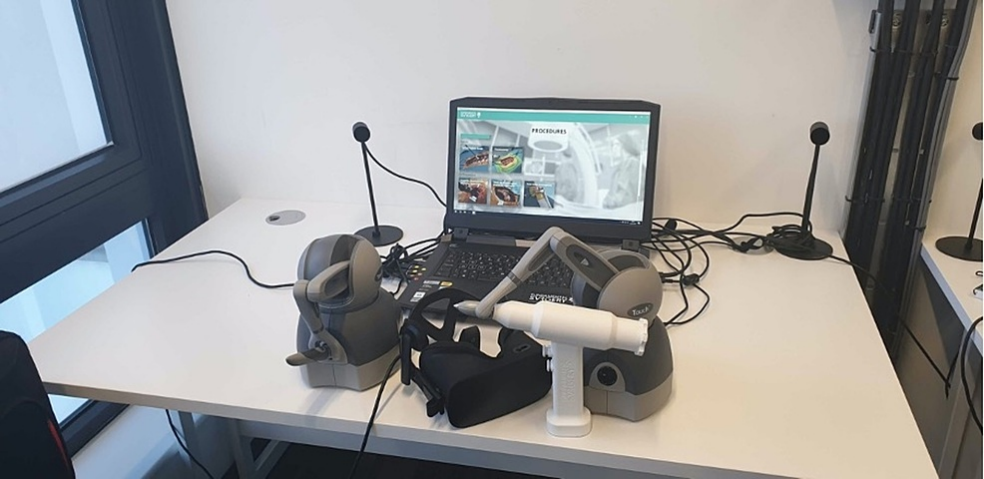
Virtual reality tool including a display device, headset and haptic devices

Participants used the VR unit provided by FundamentalVR (London, United Kingdom) to take part in the study. The unit consisted of a laptop, a VR headset and two haptic arms along with the simulation software (Figure 1). Participants were assessed on their bone drilling of a synthetic tibia (Sawbones) following practice in a VR environment.

The participants were shown a short presentation explaining the drilling task and were not allowed to watch each other performing the task. Once completed, participants took turns to complete the VR calibration test where they performed numerous tasks to get them acquainted with the VR software and haptic devices.

**Figure 2:**
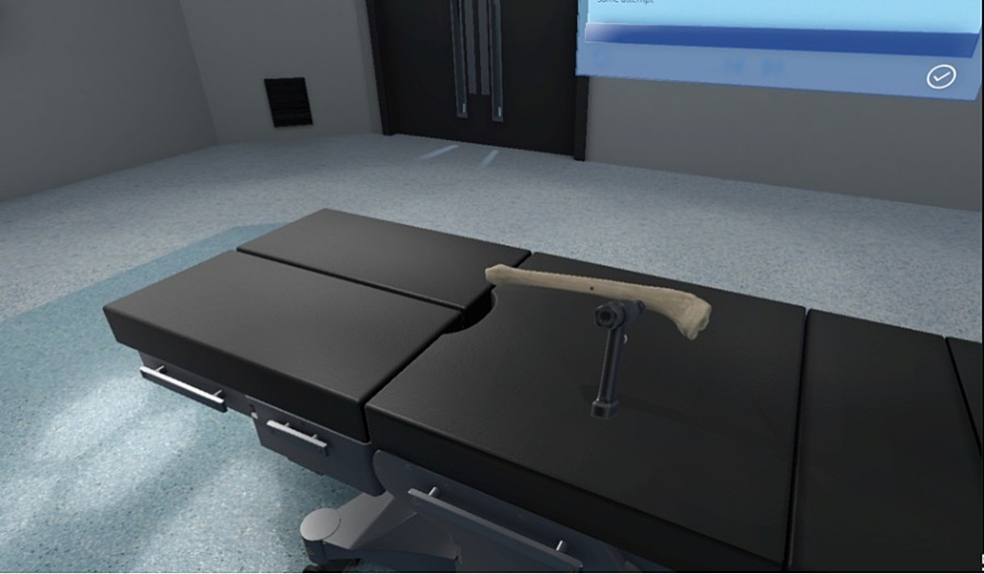
Virtual reality bone drilling software simulation

After the familiarisation part of the study was completed, participants were then randomly allocated into two groups using block randomisation. One group would perform bone drilling in a VR environment with haptic feedback, and the other group would perform the same task without haptic feedback. Audio and visual simulations were identical in both groups. In the VR-simulated environment, participants were allowed to drill a same-sided tibia bone five times before ending the session - each attempt was made in a different area along the tibial shaft (Figure 2).

**Figure 3:**
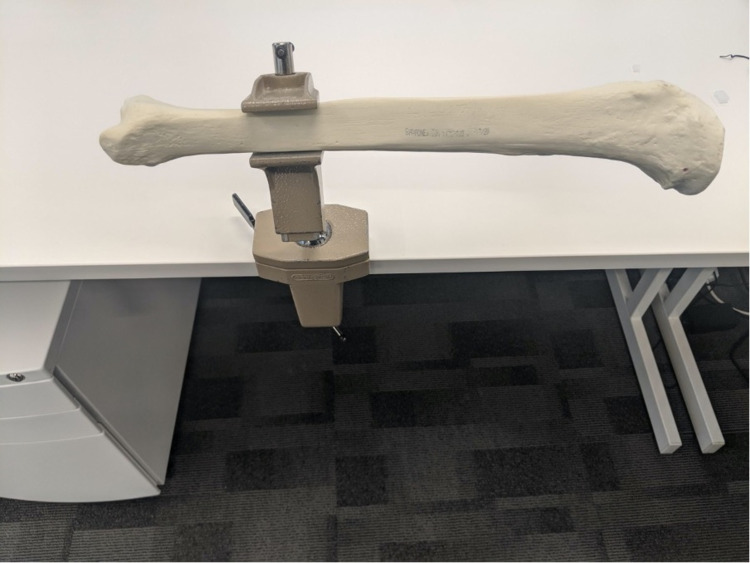
Long bone in clamp

**Figure 4:**
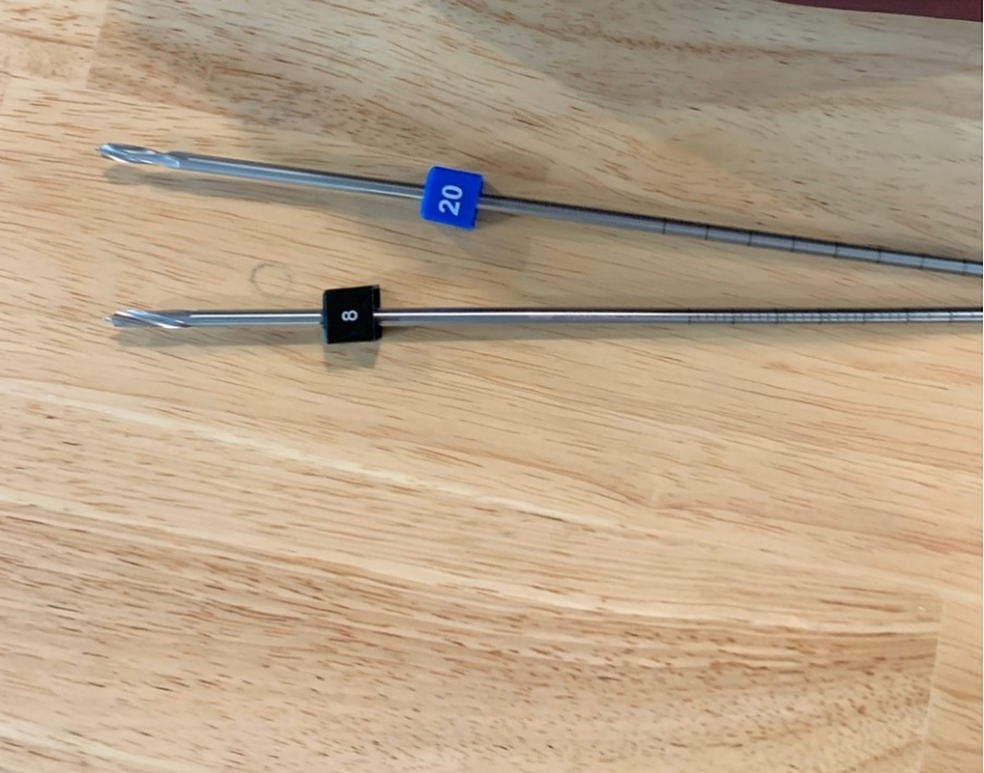
Picture of the drill bit and marker

After completing the VR session, participants were then allowed to drill a model tibia bone with a standard handheld drill (Figures 3, 4) (Video 1). The bone was held in place by a clamp. Successful attempts were recorded when the drill had penetrated the far cortex of the bone. To measure the length of the drill bit that had penetrated the bone, a marker device was placed at a neutral position on the drill bit. As the drill bit advanced into the bone, this marker would then be pushed further away from the drill tip. A measurement was then taken of the distance between the drill tip and the marker device. The width of the bone drilled was then measured using a depth gauge. The plunge distance was calculated by subtracting the measurement of the bone width from the measurement of the drill tip to a measuring device. The assessor performing the measurements was blinded to the randomisation of the participants.

**Video 1:** Participant drilling tibia sawbone following virtual reality simulation

Stimuli

The audio and visual stimuli was provided by the head-mounted display via the Oculus Rift VR device. Whilst in the VR simulation, participants were able to see their drill and tibia bone. The drilling sounds used in the simulation were recordings. The drill sounds changed based on where the bone drilling occurred - inside or outside the bone in addition to when the cortex was breached. 

Haptic feedback 

Feedback was generated from the haptic device once the participants touched the tibia bone in the VR environment. When the bone is drilled through, the haptic arm generates a force to push back against the users holding the haptic device. This force is fixed against the force applied by the participant. When the far cortex of the VR tibia bone is breached, there is less force applied from the haptic device.

Statistical analysis 

The mean drill depth for each participant was recorded, and the p-value was calculated using an independent t-test. Qualitative data analysis also took place on the recorded answers to the pre-study questionnaires for each participant.

## Results

The mean plunge distance was calculated for each synthetic tibia drilling attempt that took place. Table 3 demonstrates the actual and mean drilling depth of each participant who had performed simulated bone drilling with haptic feedback as part of their VR simulated learning, whilst Table 4 demonstrates the actual and mean drilling depth values of participants who performed simulated bone drilling without haptic feedback. The four participants who received haptic feedback simulation (mean = 46.25mm; median = 46mm; SD = 7.93) compared to the four participants in the group who received no haptic feedback during simulation (Mean = 79mm, Median = 68mm, SD = 44.23) demonstrated no statistically significant difference between the plunge depths when drilling a synthetic tibia (t(6) = -1.46; p = 0.1952).

**Table 3:** Mean drilling plunge distance (mm) for participants who experienced haptic feedback in simulation

	Plunge Depth (mm)					Average Plunge Depth (mm)
Participant 1	30	80	60	30	20	44
Participant 2	90	50	30	40	30	48
Participant 3	20	25	40	50	50	37
Participant 4	70	4	50	80	40	56

**Table 4:** Mean drilling plunge distance (mm) for participants who did not experience haptic feedback in simulation

	Plunge Depth (mm)					Average Plunge Depth (mm)
Participant 5	40	20	40	70	50	44
Participant 6	230	110	110	110	120	136
Participant 7	130	90	60	70	110	92
Participant 8	40	46	40	40	40	44

Further analysis took place on the first drilling attempt of each participant. Within the group that received haptic feedback simulation, the mean plunge distance on the first attempt was 52.5mm, and within the non-haptic group it was 110mm.

**Figure 5:**
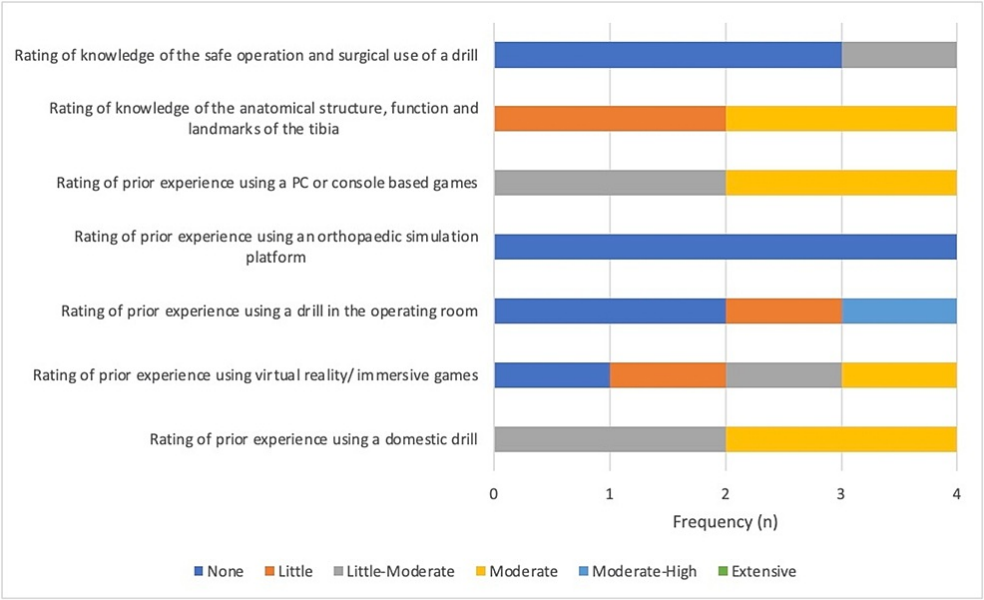
Haptic feedback participants' ratings of their experience with various tasks

**Figure 6:**
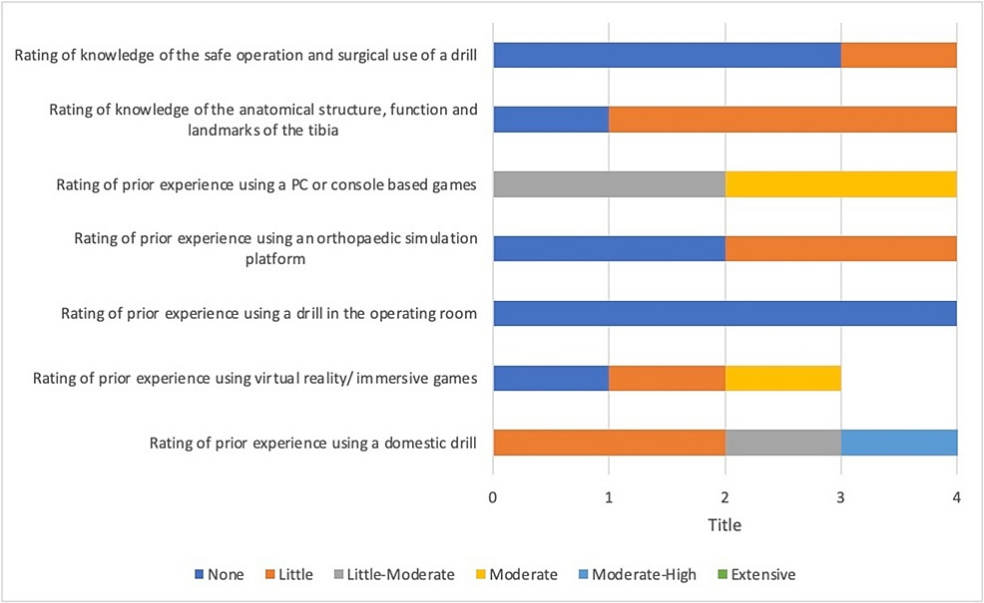
Non-haptic feedback participants’ ratings of their experience with various tasks

We also looked at the results of the self-scoring pre-study questionnaire. Participants rated their experience with prior activities and rated answers as None, Little, Little-Moderate, Moderate, Moderate-High and Extensive. Figure 5 shows the rating of participants who received haptic feedback during the virtual bone drilling simulation and Figure 6 shows the rating of participants who did not receive haptic feedback during the virtual bone drilling simulation.

## Discussion

The bone drilling study looked at the differences in plunge distance after drilling a synthetic tibia (Sawbones). Participants were medical students, junior doctors and biomedical scientists. Participants were split into two groups, with each group performing bone drilling in a VR simulated learning environment: one group received haptic feedback during their simulation and the other group did not. Although there was no statistically significant difference between the plunge depth of each group, the data suggest that the participants who received VR-simulated training feedback performed better with bone drilling. Specifically, these participants had a lower mean plunge distance than the group that did not receive haptic feedback during the VR simulation. The reduction in plunge distance in the first drill attempt between the groups was also found to be different.

It is important to highlight the importance of a 33mm difference in mean plunge distance between the groups. Asadollahi et al. [15] looked at the iatrogenic risk to the superficial femoral artery during femoral fixation. They found that the artery can be 8mm from the tip of the ideal plate screw position. Greater control of the drill obtained through haptic feedback training could mean that important structures within the operative area will be at less risk of damage. Our findings support the hypothesis that learning to drill bone in a simulated learning environment with haptic feedback will improve the motor skills needed for successful bone drilling and will contribute to training surgeons to keep structures safe. This is a skill that is advantageous for training as it produces safer practice.

Coles et al. [16] looked at the role of haptics in medical and surgical training simulators. They concluded that in the future, haptic technologies will have an important role in maintaining the skill competency for doctors and reducing the need to train on patients.

The findings in this study also corroborate other literature regarding motor learning which indicates that haptics, in conjunction with other forms of sensory feedback, are useful in the early acquisition of complex motor skills [17,18].

A study performed by Grant et al. [19] examined haptic feedback in VR in order to see if it led to an improved motor performance in participants. They found that participants who only received auditory stimuli without haptic stimulation had worse motor performance than those who experienced haptic feedback.

In our study, there were limitations which affected the results. The study size was affected by the recruitment of healthcare professionals. Due to their busy work schedule, recruitment was difficult and therefore the study included a less number of participants. In the future, multi-centre studies will be performed in the United Kingdom and the United States with a larger number of participants.

Finally, an exploration of the full potential of haptic feedback simulation teaching in accelerating motor skill acquisition is needed. This study has given us an insight into the expected benefit of the device in surgical training. The study has been performed with participants who had none to little experience of using a drill within the operating room. The improvement in drill control within novices is clear from our study and the potential benefit on already skilled surgeons is an area for further research.

## Conclusions

The ability to reduce plunge distance was improved when participants performed a training simulation with a VR haptic feedback device compared to without a haptic feedback device. The data indicate that the incorporation of VR simulation training into the surgical curriculum should be considered in order to maximise the potential of surgical trainees due to limited learning opportunities. The long-term effects of teaching delivered by VR simulators are unknown, and therefore there is further scope for research in this area.
